# A Chromosome-Level Genome Assembly and Annotation of the Chinese Porcupine (*Hystrix hodgsoni*) Reveals the Expansion of Olfactory-Related Gene Families

**DOI:** 10.3390/genes17060596

**Published:** 2026-05-22

**Authors:** Nannan Chen, Jianxuan Zhou, Xinjie Liu, Meidong Jing, Libo Jiang, Fengtang Yang

**Affiliations:** 1 School of Life Sciences and Medicine, Shandong University of Technology, Zibo 255049, China; 23410011011@stumail.sdut.edu.cn (N.C.); 23410010998@stumail.sdut.edu.cn (J.Z.); 25410011042@stumail.sdut.edu.cn (X.L.); 2 College of Life Sciences, Nantong University, Nantong 226019, China

**Keywords:** Chinese porcupine, *Hystrix hodgsoni*, chromosome-level genome, comparative genomics, gene family expansion, olfactory genes

## Abstract

Background/Objectives: The Chinese porcupine (*Hystrix hodgsoni*) is a distinctive rodent species characterized by specialized ecological adaptations and sensory traits; however, genomic resources for this species have remained limited. This study aims to provide a reliable reference for comparative and evolutionary analyses by constructing a high-quality genome. Methods: We generated a chromosome-level genome assembly of the Chinese porcupine using long-read sequencing combined with chromatin conformation-based scaffolding, followed by comprehensive structural and functional annotation. Comparative genomic analyses across representative mammals and functional enrichment analyses were conducted to investigate lineage-specific gene family dynamics. Results: The assembled genome shows high contiguity and completeness. Comparative analyses revealed a substantial number of gene families significantly expanded along the porcupine lineage. Functional enrichment demonstrated strong overrepresentation of olfactory-related processes, including olfactory receptor activity, odorant binding, and detection of chemical stimuli. Additionally, several expanded families were associated with epidermal differentiation, keratinization, and skin development. Conclusions: Gene family expansions in the Chinese porcupine are biased toward sensory perception and epidermal functions, suggesting potential genetic bases for its enhanced environmental sensing and integumentary specialization. This assembly provides an important genomic resource for porcupine research and new insights into the molecular mechanisms underlying sensory and skin-related adaptations in rodents.

## 1. Introduction

The Chinese porcupine (*Hystrix hodgsoni*) is a large, nocturnal rodent that belongs to the family Hystricidae within the order Rodentia. It is widely distributed across South and Southeast Asia, encompassing southern China, Southeast Asia [1], and the Himalayas region [2,3]. As one of the most morphologically distinctive porcupine species in Asia, H*. hodgsoni* is recognized for its robust physique and prominent, highly specialized quill structures [4], which represent striking features of its morphological and ecological adaptation [3].

Ecological studies have demonstrated that porcupines play a crucial role in ecosystems by acting as both seed dispersers and ecosystem engineers [5], significantly influencing forest regeneration processes and soil dynamics [3,6]. Furthermore, their highly specialized quill structure serves as an effective defense mechanism, making porcupines an ideal model for investigating mammalian morphological innovation and adaptive evolution [7,8]. However, due to habitat loss and disturbances caused by human activities, porcupine populations have declined in certain regions [8,9], highlighting the importance of understanding their adaptive traits from genetic and genomic perspectives.

Although porcupines are of considerable ecological and evolutionary interest, their genetic basis remains insufficiently characterized at a genome-wide scale [10]. Previous studies have primarily concentrated on the morphology, behavior, and conservation biology of porcupines [6,8,11]. For example, Mori et al. investigated the mechanical properties of porcupine quills and found that they exhibit exceptional fracture resistance [12]. Subsequent developmental studies have suggested that keratin and keratin-associated proteins play critical roles in quill formation [13,14]. Nevertheless, the molecular regulatory mechanisms and genetic architecture underlying this highly specialized epidermal phenotype have not yet been systematically investigated using whole-genome approaches.

Beyond epidermal specialization, olfactory perception represents another key component of porcupine ecological adaptation. Research has shown that the olfactory receptor (OR) gene family in porcupines may play an important role in their sensory adaptation and ecological functions [15]. Studies have indicated that odorant-binding proteins (OBPs) in porcupine nasal tissue are involved in odor perception [16,17,18]. Despite these findings, systematic analyses of the porcupine olfactory genome remain scarce, particularly regarding the expansion patterns of OR gene families and their potential association with ecological adaptation.

In addition, population genetic analyses of *Hystrix* species have revealed relatively low levels of genetic diversity under conditions of habitat fragmentation [3]. This finding highlights the urgent need to develop high-quality genomic resources to assess their genetic health and evolutionary potential. Comprehensive genetic and genomic studies are therefore essential for elucidating adaptive mechanisms, reconstructing evolutionary history, and formulating effective conservation strategies for porcupines.

Cytogenetic studies have demonstrated pronounced karyotypic diversity among species of the genus Hystrix. For example, the crested porcupine (*Hystrix cristata*) exhibits notable chromosomal number variation across different geographical populations. In some African populations, the diploid chromosome number of *H. cristata* is 2*n* = 54 [19], whereas in other populations it is 2*n* = 60 [20,21]. In contrast, the Malayan porcupine (*Hystrix brachyura*) has a significantly different karyotype structure, with a diploid chromosome number of 2*n* = 66 [22]. Chromosomal number and structural variation among closely related species reflect a high degree of evolutionary plasticity within this lineage. However, due to the lack of high-quality reference genomes, the mechanisms underlying chromosomal evolution in porcupines remain poorly understood.

The phylogenetic relationships between porcupines and other rodent lineages have long been debated [23]. Traditional morphological classifications and analyses based on limited molecular markers have failed to fully resolve the evolutionary relationships between the genus *Hystrix* and other rodent groups, such as guinea pigs [24,25]. In recent years, comparative studies based on whole-genome data have gradually helped to address this research gap [26,27] and provided new perspectives on the evolutionary relationships among rodents [28]. Nevertheless, the absence of a chromosome-level reference genome for *H. hodgsoni* has constrained in-depth investigations into its evolutionary and functional biology.

With the rapid advancement of high-throughput sequencing technologies and genome assembly strategies, the generation of high-quality genomes for non-model species has become increasingly feasible [29,30,31]. To date, a draft assembly for *H. cristata* has been released (GenBank: GCA_004026905.1); however, genomic resources for *H. hodgsoni* remain limited, with only mitochondrial genome data currently available [10], which severely restricts comprehensive evolutionary and functional genomic studies.

To address this gap, we present the first chromosome-level reference genome for *H. hodgsoni*, assembled using PacBio HiFi [32] long-read sequencing combined with Hi-C [33] chromosome conformation capture technology. Based on this high-quality genome, we performed comprehensive comparative genomic analyses, with a primary focus on the evolutionary expansion of OR gene families associated with sensory adaptation. In addition, we investigated keratin gene families related to quill development and epidermal specialization, providing complementary insights into the molecular basis of porcupine morphological traits. Together, our results offer a genomic perspective on sensory adaptation and morphological evolution in porcupines, establishing a valuable resource for future functional genomics and conservation genetic studies.

## 2. Materials and Methods

### 2.1. Sample Selection and Sequencing

A healthy adult female *H. hodgsoni*, sourced from a wild population in southern China, was used for whole-genome sequencing and de novo assembly. All the experimental procedures involving animal handling and sampling in this study were in accordance with the Laboratory Management Regulations of Shandong University of Technology (No. 163, approved on 8 January 2025).

High-molecular-weight genomic DNA was isolated from hindlimb muscle tissue using the phenol–chloroform extraction method. Prior to extraction, the tissue sample was field-preserved in 100% ethanol and subsequently maintained under long-term cryopreservation to ensure nucleic acid integrity. DNA quality was evaluated by 1% agarose gel electrophoresis, and DNA concentration was quantified using a NanoDrop 2000 spectrophotometer (NanoDrop Technologies, Wilmington, DE, USA) and a Qubit 3.0 fluorometer (Life Technologies, Carlsbad, CA, USA), ensuring the sample met the stringent requirements for both long-read and short-read sequencing platforms.

For long-read sequencing, a single-molecule real-time (SMRT) library was constructed using the SMRTbell Express Template Prep Kit 2.0 (Pacific Biosciences, Menlo Park, CA, USA) with an average insert size of approximately 8 kb selected using the BluePippin system (Sage Science, Beverly, MA, USA). The HiFi library was sequenced on the PacBio Sequel II platform in circular consensus sequencing (CCS) mode. Raw PacBio subreads were processed using CCS software v6.4.0 to generate high-fidelity (HiFi) reads, retaining sequences with a minimum predicted quality score of Q20. Adapter sequences were subsequently trimmed from the HiFi reads using Cutadapt v4.6 [34].

For chromosome-scale scaffolding, genomic DNA was cross-linked with formaldehyde and extracted for Hi-C library construction using the Dovetail Omni-C Kit (Dovetail Genomics, Scotts Valley, CA, USA) following the manufacturer’s standard protocol. The Hi-C library was paired-end sequenced (150 bp) on the Illumina NovaSeq 6000 platform (Illumina, Inc., San Diego, CA, USA). The raw Hi-C data were filtered using fastp v0.19.4 [35] to remove adapter sequences, low-quality reads (Phred quality < Q15), and reads shorter than 30 bp.

All library construction and sequencing, including PacBio HiFi and Hi-C, were conducted by Berry Genomics (Beijing, China). A summary of the sequencing libraries and platforms is provided in Table S1.

### 2.2. Genome Size Estimation and Assembly

The genome size of *H. hodgsoni* was estimated using a *k*-mer frequency-based method with quality-controlled PacBio HiFi reads. The *k*-mer counting was performed using Jellyfish v2.2.10 [36] with the parameters “-C -s 10000000000 -t 80”, where the hash size was set sufficiently large to accommodate the estimated genome size and sequencing depth. A series of *k*-mer sizes (*k* = 21, 23, 25, 27, 29, 31, 33, 35, 37, and 39) were applied to evaluate the consistency and stability of genome size estimation across different scales. The resulting *k*-mer frequency distributions were used as input for GenomeScope v2.0 [37] and findGSE v1.94 [38] to estimate the haploid genome size, heterozygosity rate, and repeat sequence content (Table S2).

In addition, genome size was independently calculated using a formula proposed by Li and Waterman [39], which is based on the relationship between sequencing depth and *k*-mer coverage depth. The equation used to calculate the total number of *k*-mers is:

(1) $$M=N\times (\frac{L-K+1}{L})$$

where *N* represents the total number of sequenced bases (Q20 yield), *L* denotes the average read length of HiFi reads, *K* is the *k*-mer size, and *M* corresponds to the total number of generated *k*-mers. The genome size was then estimated by dividing *M* by the peak *k*-mer coverage depth identified from the *k*-mer frequency distribution. Genome size estimates derived from different *k*-mer sizes and analytical approaches were compared to ensure robustness and reliability.

We constructed a chromosome-scale genome assembly of *H. hodgsoni* using PacBio HiFi and Hi-C sequencing data. Initially, PacBio HiFi reads were assembled into contigs using hifiasm v0.20.0-r639 [40] with default parameters. Redundant haplotypic sequences were subsequently removed using purge_dups v1.2.5 [41]. The filtered Hi-C paired-end reads were then aligned to the primary contig assembly using BWA-MEM v0.7.18-r1243-dirty [42]. Uniquely mapped valid read pairs with a minimum mapping quality of 40 were extracted using pairtools v1.0.2 [43]. Chromosome-scale scaffolding, including clustering, ordering, and anchoring of contigs, was performed using YaHS v1.2a.1 [44] based on the valid Hi-C read pairs. Hi-C contact maps were visualized and manually curated using Juicebox v1.8.8 [45] and HiCExplorer v3.5.1 [46] to identify and correct potential misassemblies. Finally, remaining gaps in the assembly were closed using TGS-GapCloser v1.0.1 [47] with PacBio HiFi reads to further improve assembly continuity.

### 2.3. Genome Quality Evaluation

To comprehensively evaluate the quality of the *H. hodgsoni* genome assembly, we assessed assembly completeness, base accuracy, functional completeness, and chromosome-scale scaffolding status. Assembly completeness was evaluated by aligning PacBio HiFi reads to the assembled genome using minimap2 v2.17 [48], and the mapping rate, genome coverage, and average sequencing depth were calculated with SAMtools v1.19.2 [49]. Base accuracy was assessed by calculating the consensus quality value (QV) and k-mer completeness using Merqury v1.330 [50] based on quality-controlled PacBio HiFi reads. Functional completeness of the genome assembly was evaluated using BUSCO v5.8.0 [51] in genome mode with the mammalia_odb10 dataset.

For chromosome-scale assessment, Hi-C data were used to scaffold contigs into chromosome-level assemblies, resulting in 33 pseudo-chromosomes under a diploid model. Since the sample was from a female individual, no Y chromosome was detected. The X chromosome was identified based on previously reported cytogenetic karyotype information of *H. hodgsoni* [52] and whole-genome alignment to the human reference genome (GRCh38) [53] using MUMmer v4.0 [54].

### 2.4. Gene Prediction and Functional Annotation

#### 2.4.1. Identification of Non-Coding RNAs

In the genome of *H. hodgsoni*, three classes of non-coding RNAs were identified, including transfer RNAs (tRNAs), ribosomal RNAs (rRNAs), and microRNAs (miRNAs). Transfer RNAs (tRNAs) were annotated using tRNAscan-SE v1.23 [55] with the organellar search mode and a Cove cut-off score of 15 to infer their secondary structures. Ribosomal RNA (rRNA) genes were predicted using barrnap v0.9 [56] with default parameters. MicroRNAs (miRNAs) and other small non-coding RNAs were identified using cmscan v1.1.3 [57] from the Infernal package, based on the Rfam v14.8 database [58].

Repeat prediction. Repetitive elements in the *H. hodgsoni* genome were annotated using a combination of de novo and homology-based approaches. First, RepeatModeler v2.0.2 [59] was employed for de novo identification of repetitive sequences, integrating multiple tools including RECON, RepeatScout, TRF, LTRharvest, and LTR_retriever. The predicted repeat sequences were manually curated by comparison with Repbase v19.06 database [60], and a custom repeat library was constructed. This custom library, together with the Dfam consensus database and Repbase library, was further classified into different repeat families using the PASTEClassifier v1.0 [61] script of REPET v2.5. Finally, RepeatMasker v4.1.2 [62] was used to mask repetitive sequences in the genome assembly based on the combined repeat libraries.

#### 2.4.2. Protein-Coding Gene Prediction

To annotate protein-coding genes in the *H. hodgsoni* genome, we employed ANNEVO v2.2 [63], a deep learning-based ab initio gene prediction tool. Gene prediction was performed on the repeat-masked genome assembly using a pre-trained mammalian model (ANNEVO_Mammalia.pt), which was specified via the “--model_path” option. The initial gene models generated by ANNEVO were filtered based on an annotation edit distance (AED) < 0.5 and a minimum protein length of more than 10 amino acids. Subsequently, the predicted gene models were further curated using AGAT v1.2.0 [64] to remove (1) coding sequences (CDSs) < 300 bp, (2) CDSs not divisible by three, and (3) gene models with premature stop codons. The remaining high-confidence gene models were retained for downstream analyses.

#### 2.4.3. Functional Annotation

The predicted protein sequences from the *H. hodgsoni* genome were functionally annotated using multiple public databases. First, protein sequences were aligned against the NCBI non-redundant (NR) protein database and the Animal Transcription Factor Database using BLASTP v2.2.30 [65], with an E-value threshold of 1e-5. Additional functional annotations were obtained by aligning protein sequences to the UniProtKB database using DIAMOND v2.0.11.149 [66] in sensitive mode. Protein domains and conserved motifs were identified using InterProScan v5.53-87 [67], based on multiple integrated databases, including Pfam, PANTHER, and TIGRFAMs. Further functional annotation was performed using eggNOG-mapper v2 [68] for Gene Ontology (GO) and Kyoto Encyclopedia of Genes and Genomes (KEGG) pathway annotations, based on the eggNOG v5.0 database [68].

### 2.5. Comparative Genomics Analysis

#### 2.5.1. Orthogroup Inference and Phylogenetic Framework Construction

To investigate gene family evolution in the Chinese porcupine (*H. hodgsoni*), a comparative genomic analysis was conducted using four representative mammals, including human (*Homo sapiens*), mouse (*Mus musculus*), African hedgehog (*Atelerix albiventris*), and guinea pig (*Cavia porcellus*), resulting in a dataset of five species. To avoid artifacts in gene family dynamics analyses caused by fragmented assemblies, our comparative framework strictly incorporated species with high-quality reference genomes. Within this dataset, *H. sapiens* and *M. musculus* were included as outgroup baselines, *C. porcellus* represented a related rodent lineage, and *A. albiventris* provided an ecological comparison. For each gene, only the longest protein-coding transcript was retained as the representative sequence. Orthologous groups (orthogroups) were inferred from the predicted proteomes of the five species using OrthoFinder v2.3.8 [69,70] with DIAMOND sequence alignment (parameter: -S diamond). The distribution patterns of shared and species-specific orthogroups were visualized using an UpSet plot.

A rooted species tree inferred by OrthoFinder based on single-copy orthologs (SpeciesTree_rooted.txt) was used as the phylogenetic framework for downstream gene family evolution analyses. Given the limited taxon sampling, this tree was primarily used to support comparative modeling rather than for detailed phylogenetic inference.

#### 2.5.2. Gene Family Expansion and Contraction Analysis

To characterize gene family size evolution along the Chinese porcupine lineage, gene copy number matrices inferred by OrthoFinder were used as input for CAFE (Computational Analysis of gene Family Evolution) v5.1.0 [71]. CAFE applies a stochastic birth–death model to estimate gene gain and loss processes along phylogenetic branches while accounting for species relationships and branch lengths. Prior to analysis, gene families with more than 100 copies in any species were excluded to minimize potential annotation artifacts. In addition, only gene families present in at least two species were retained to improve statistical robustness.

Under the default single-rate (λ) model, global rates of gene family expansion and contraction were estimated. Likelihood ratio tests (LRTs) implemented in CAFE were applied to assess significant gene family size changes along specific branches, with a significance threshold of *p* < 0.05. Gene families showing significant expansion or contraction along the Chinese porcupine lineage were extracted for subsequent functional annotation and structural characterization.

#### 2.5.3. Functional Enrichment Analysis of Expanded Gene Families

To further investigate the functional characteristics of gene families expanded in *H. hodgsoni*, gene families showing significant expansion along the porcupine lineage identified by CAFE analysis were ranked according to their expansion magnitude. The top 20 expanded gene families were selected for subsequent functional enrichment analyses. Protein sequences corresponding to these gene families were extracted for downstream analyses.

GO [72,73] enrichment analysis was conducted to assess functional enrichment across three categories: Biological Process, Molecular Function, and Cellular Component. In parallel, KEGG [74] pathway enrichment analysis was performed to identify biological pathways associated with the expanded gene families. Functional enrichment analyses were carried out using the clusterProfiler R package v4.16.0 [75], with all annotated protein-coding genes in the *H. hodgsoni* genome used as the background gene set. Multiple testing correction was performed using the Benjamini–Hochberg method, and terms with a false discovery rate (FDR) < 0.05 were considered statistically significant.

Chromosomal distribution and density analysis of expanded OR gene families. Based on the gene family expansion analysis, four OR gene families were selected from the top 20 significantly expanded gene families along the *H. hodgsoni* lineage for further chromosomal distribution analysis. Gene IDs belonging to each OR orthogroup were retrieved according to OrthoFinder classification results and mapped to genomic coordinates using the *H. hodgsoni* genome annotation file.

To evaluate large-scale chromosomal distribution patterns, the genome was partitioned into non-overlapping 1-Mb windows, and the number of OR genes within each window was calculated. Genomic regions showing significantly elevated OR gene density were identified as candidate clustering hotspots. For chromosomes exhibiting strong OR enrichment (notably chromosome 9), detailed gene-level visualization was further performed to examine local gene arrangement patterns and assess whether gene family expansion was driven by tandem duplication events, as indicated by dense gene clusters with consistent orientation.

## 3. Results

### 3.1. Genome Assembly and Evaluation

The genome size of the *H. hodgsoni* was estimated using three independent methods across ten different *k*-mer sizes, resulting in values ranging from 2.19 Gb to 2.65 Gb (Table S1). Genome-wide heterozygosity estimates ranged from 0.61% to 0.80%, indicating a moderately heterozygous genome.

**Figure 1:**
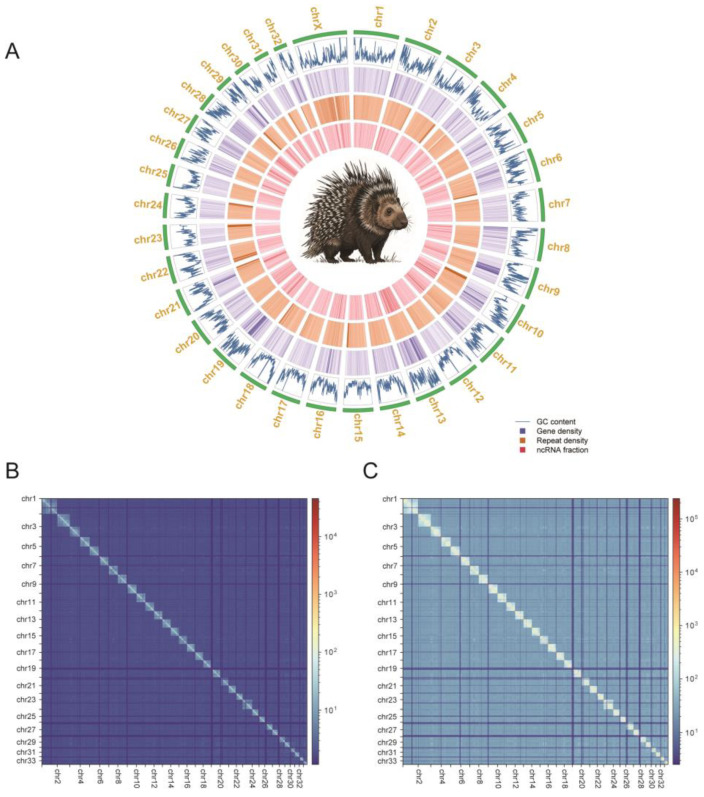
Chromosome-level genome features and Hi-C contact maps of the Chinese porcupine (*Hystrix hodgsoni*). (**A**) Circos plot showing genome-wide features across 33 pseudochromosomes. From outer to inner rings: GC content, gene density, repeat density, and ncRNA distribution, calculated using sliding windows along each chromosome. (**B**) Genome-wide Hi-C interaction heatmap at 500 kb resolution, illustrating large-scale chromosomal contact patterns across all pseudochromosomes. (**C**) Genome-wide Hi-C interaction heatmap at 100 kb resolution, revealing finer-scale chromosomal interaction structures and further supporting the continuity and accuracy of chromosome-level scaffolding. Color intensity indicates normalized contact frequency on a logarithmic scale.

An initial contig-level assembly was generated using 159 Gb (~27×) of PacBio HiFi long reads and 158 Gb (~100×) of Hi-C short-read data (Table S2), producing a primary assembly of 2.67 Gb and a contig N50 of 39.73 Mb (Table 1). Using high-confidence Hi-C interaction pairs, contigs were further anchored, ordered, and oriented with YaHS, resulting in a chromosome-scale assembly comprising 33 pseudochromosomes. This chromosome number is consistent with previously reported karyotype analyses of *H. hodgsoni*. As the sampled individual was female, no Y chromosome was detected. The X chromosome was identified based on whole-genome alignment of the primary assembly to the human reference genome (GRCh38) using MUMmer together with published cytogenetic evidence for *H. hodgsoni* [52], and the comprehensive genomic landscape is visualized in Figure 1A.

Hi-C sequencing generated a total of 1.28 billion clean paired-end reads, of which 67.01% were successfully mapped to the assembled genome, and 749.68 million non-duplicated read pairs were retained for downstream scaffolding (Table S3). Hi-C contact heatmaps generated at 500 kb and 100 kb resolutions showed strong and continuous intrachromosomal interaction signals along the diagonal of each pseudochromosome, accompanied by markedly reduced interchromosomal interactions, indicating high structural accuracy and reliability of the chromosome-level assembly (Figure 1B,C).

After chromosome-level scaffolding, the final assembly had a total size of 2.36 Gb and consisted of 33 scaffolds, with a scaffold N50 of 74.88 Mb. The largest scaffold corresponded to chromosome X and spanned 135.49 Mb (Table 1; Table S4). The assembled genome size was consistent with the genome size estimated by findGSE at k = 31 (Table S1). The average GC content of the *H. hodgsoni* genome was 41.70%, which is comparable to that of other rodent species, including *M. musculus* (41.80%), Rattus norvegicus (41.70%), and *C. porcellus* (42.00%) (Figure 1A; Table 1).

Genome assembly completeness was evaluated by mapping PacBio HiFi reads back to the assembled genome, resulting in a read alignment rate of 99.99% and a genome coverage of 99.99%, indicating high assembly completeness. Base-level accuracy assessment using Merqury yielded a consensus base pair QV of 69.15, a *k*-mer completeness of 92.48%, and an estimated error rate of 1.21e-07. Functional completeness was further assessed using BUSCO with the mammalia_odb10 dataset, showing that 98.30% of conserved genes were completely recovered, including 96.70% single-copy and 1.60% duplicated BUSCOs (Table S5).

### 3.2. Genome Annotation and Gene Content

Based on the chromosome-level genome assembly, a comprehensive genome annotation was performed for *H. hodgsoni* to characterize its gene content and genomic structural features. In total, 203 ribosomal RNA (rRNA), 14,270 transfer RNA (tRNA), 437 microRNA (miRNA), and 2,949 small nuclear RNA (snRNA) genes were identified (Table S6). These non-coding RNA genes were widely distributed across the genome without apparent chromosomal bias, and their spatial distribution generally corresponded to gene-dense regions (Figure 1A).

Repetitive elements accounted for a substantial proportion of the *H. hodgsoni* genome, with a total length of approximately 841.42 Mb, representing 35.67% of the assembled genome (Figure 1A). Among different repeat classes, retrotransposons were the most abundant, comprising 26.49% of the genome, including long interspersed nuclear elements (LINEs, 15.15%) and short interspersed nuclear elements (SINEs, 4.35%). Long terminal repeat (LTR) retrotransposons constituted 6.99% of the genome and were mainly composed of retroviral-related elements. DNA transposons accounted for 3.45% of the genome, primarily belonging to the Tc1-IS630-Pogo and hobo-Activator families. In addition, 4.33% of repetitive sequences could not be assigned to known repeat families. Small RNAs, satellite sequences, simple repeats, and low-complexity regions contributed 0.10%, 0.22%, 0.91%, and 0.21% of the genome, respectively (Table S7). Comparative analysis indicated that the overall repeat content of the *H. hodgsoni* genome (35.67%) was markedly lower than that of *A. albiventris* (58.19%), but was comparable to that observed in other small mammalian species.

A total of 23,424 protein-coding genes were predicted in the *H. hodgsoni* genome, with an average gene length of 32.67 kb and a mean of 9.5 exons per gene (Table 1). BUSCO assessment in transcriptome mode showed that the predicted gene set achieved a completeness of 95.80%, indicating a high-quality gene annotation (Table 1). The observed reduction in BUSCO completeness compared to the assembly level (98.30%) is a direct consequence of the strict filtering criteria (e.g., removal of incomplete or truncated models) applied during the gene prediction pipeline to ensure protein set reliability. Functional annotation revealed that 22,525 genes (96.16%) were assigned at least one functional annotation based on searches against public databases, including NR, UniProtKB, InterPro, and eggNOG. Among these databases, eggNOG provided the highest annotation coverage (95.72%), followed by UniProtKB (92.93%) and InterPro (91.26%) (Table S8).

The chromosomal distribution of protein-coding genes was generally proportional to chromosome length and local gene density, and no pronounced gene deserts or extremely gene-rich regions were detected across chromosomes (Table S4).

### 3.3. Comparative Genomics and Gene Family Evolution

To clarify the phylogenetic position of *H. hodgsoni* and to investigate patterns of gene family evolution, a comparative genomic analysis was performed using protein sequences from five representative mammalian species: *H. hodgsoni*, *H. sapiens*, *M. musculus*, *A. albiventris*, and *C. porcellus*. Orthologous groups were inferred using OrthoFinder, resulting in the identification of 18,928 orthogroups across the five species (Table S9).

Of the 23,424 predicted protein-coding genes in the *H. hodgsoni* genome, 21,352 genes (91.20%) were assigned to orthogroups, whereas 2,072 genes (8.80%) were not assigned. In total, 16,934 orthogroups contained at least one *H. hodgsoni* gene. Only 99 orthogroups were identified as species-specific to *H. hodgsoni*, comprising 367 genes (1.6% of all protein-coding genes), indicating that the majority of genes in the *H. hodgsoni* genome are evolutionarily conserved among mammals.

**Figure 2:**
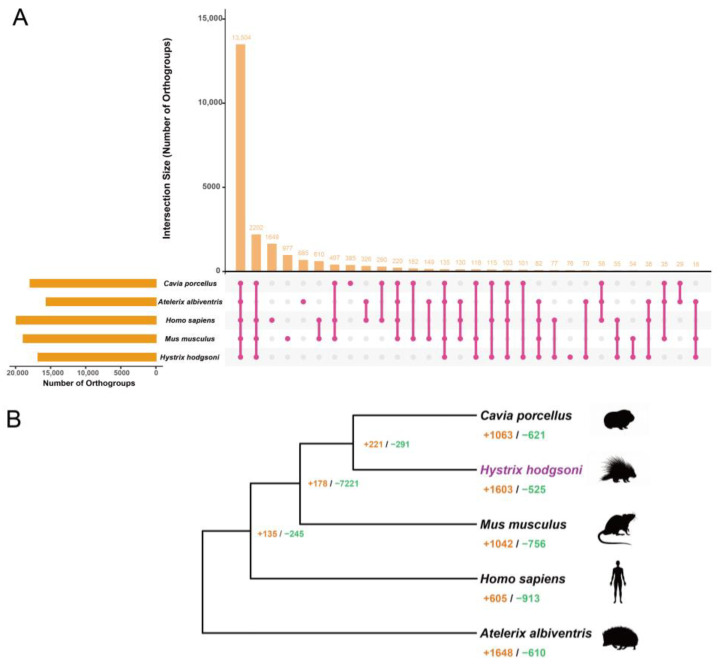
Comparative genomic analysis and gene family dynamics across five mammalian species. (**A**) UpSet plot illustrating the distribution and overlap of shared and species-specific orthogroups based on OrthoFinder analysis. (**B**) Phylogenetic tree depicting the expansion (orange, +) and contraction (green, −) of gene families across lineages as inferred by CAFE.

The UpSet analysis (Figure 2A) illustrated the distribution patterns of shared and species-specific orthogroups among the five species, highlighting both the conserved core gene sets and lineage-specific gene family components. In addition, a total of 5,894 single-copy orthologous groups were identified among the five species and used to reconstruct their phylogenetic relationships (Figure 2B). The resulting species tree showed that *H. hodgsoni* clustered most closely with *A. albiventris*, which is consistent with their established taxonomic classification.

To further investigate gene family evolution, the gene family size matrix generated by OrthoFinder was analyzed using CAFE. Among gene families showing significant changes across the phylogeny (*p* < 0.05), 177 families exhibited significant expansion, whereas 138 families showed significant contraction along the *H. hodgsoni* lineage (Figure 2B; Table S10). Distinct patterns of gene family expansion and contraction were observed across different branches of the phylogenetic tree, reflecting lineage-specific gene gain and loss events during mammalian evolution.

**Figure 3:**
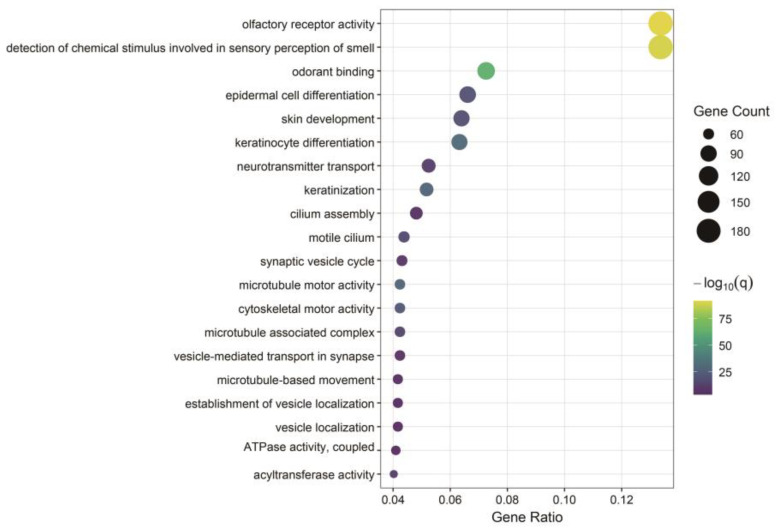
Gene Ontology (GO) enrichment analysis of significantly expanded gene families in the *Hystrix hodgsoni* lineage. The x-axis represents the gene ratio. The length of the bars indicates the number of enriched genes (Gene Count), and the color gradient reflects statistical significance. The *q*-value corresponds to the false discovery rate (FDR) adjusted via the Benjamini–Hochberg procedure.

**Figure 4:**
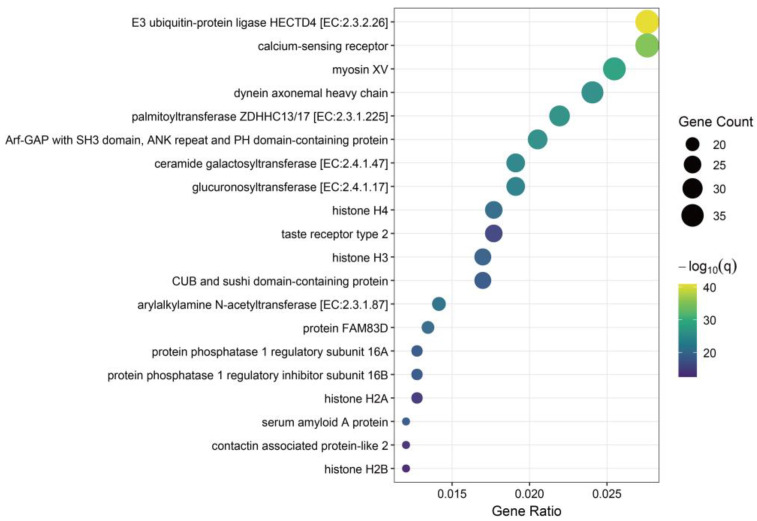
Kyoto Encyclopedia of Genes and Genomes (KEGG) pathway enrichment analysis of significantly expanded gene families in the *Hystrix hodgsoni* lineage. The x-axis represents the gene ratio. The length of the bars indicates the number of enriched genes (Gene Count), and the color gradient reflects statistical significance. The *q*-value corresponds to the false discovery rate (FDR) adjusted via the Benjamini–Hochberg procedure.

Functional enrichment analyses based on GO and KEGG pathways were performed for genes belonging to the top 20 most significantly expanded gene families along the *H. hodgsoni* lineage. These expanded gene families showed significant enrichment in multiple biological processes and molecular functions related to sensory perception and epidermal structure (Figure 3 and Figure 4). GO enrichment analysis further revealed that the most overrepresented terms were associated with olfactory-related functions, including OR activity, odorant binding, and detection of chemical stimulus involved in sensory perception of smell.

In addition, several biological processes related to epidermal differentiation and cornification were significantly enriched among the expanded gene families, including epidermal cell differentiation, keratinization, and skin development. KEGG pathway analysis revealed that these expanded gene families were enriched in olfactory-related pathways, as well as pathways associated with cytoskeletal organization, motor protein activity, and signal transduction. Collectively, these results indicate that gene family expansions in *H. hodgsoni* are biased toward sensory and epidermal-related functions, potentially reflecting lineage-specific adaptations and specialized phenotypic traits.

### 3.4. Chromosomal Distribution and Expansion Patterns of OR Genes

Comparative genomic analyses revealed that several OR gene families have undergone significant expansion in the *H. hodgsoni* lineage. To investigate the genomic basis of these expansion events, we analyzed the chromosomal distribution of the major expanded OR gene families.

**Figure 5:**
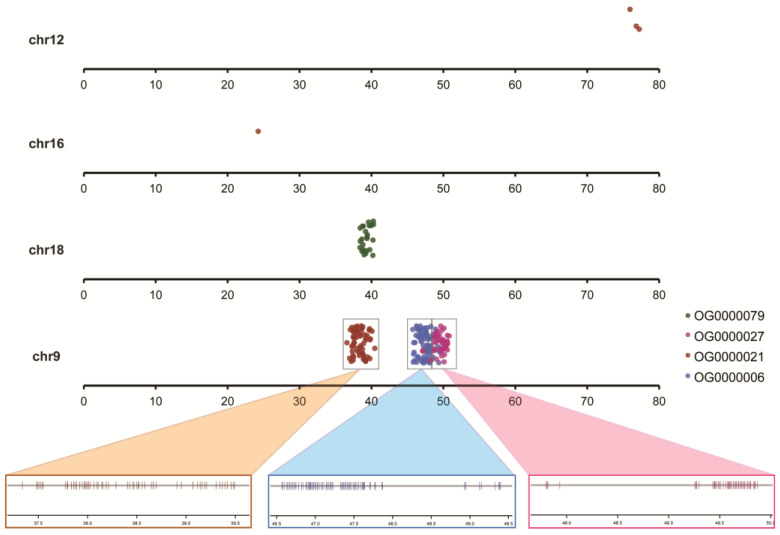
Chromosomal distribution and clustering of expanded olfactory receptor gene families in *Hystrix hodgsoni*. Genomic locations of representative expanded OR gene families (OG0000021, OG0000006, OG0000027, and OG0000079) are shown across chromosomes 12, 16, 18, and 9. Each dot represents the genomic position of an OR gene copy. Most expanded OR genes are densely clustered on chromosome 9, mainly within the region of approximately 36–52 Mb, whereas only a few scattered copies are observed on other chromosomes without obvious clustering. Enlarged views of three representative regions on chromosome 9 illustrate local gene organization patterns, showing multiple adjacent OR gene clusters belonging to different orthogroups.

OR genes exhibited a highly non-uniform distribution across the genome, rather than being randomly dispersed among chromosomes (Figure 5). Several significantly expanded OR gene families, including OG0000021, OG0000006, and OG0000027, were predominantly concentrated within local regions of chromosome 9, forming multiple dense gene clusters within approximately 36–52 Mb. In contrast, other chromosomes (such as chromosomes 12, 16, and 18) harbored only a few scattered OR gene copies and did not display obvious clustering patterns.

Fine-scale structural analysis of OR gene organization on chromosome 9 further revealed that different OR gene families formed multiple adjacent yet relatively independent gene cluster blocks (Figure 5). Within these clusters, OR genes were arranged in consecutive arrays with mixed transcriptional orientations, a genomic configuration characteristic of tandem duplication events, suggesting that local duplication played a major role in driving OR gene family expansion.

Collectively, these results indicate that expanded OR gene families in *H. hodgsoni* exhibit pronounced chromosomal clustering and tandem duplication patterns, implying that local genomic rearrangements and repeated duplication events may have contributed substantially to the evolutionary expansion of the OR repertoire in this species.

## 4. Discussion

In this study, we generated a high-quality chromosome-level reference genome for *H. hodgsoni*, providing a solid foundation for systematic investigations of its genomic architecture and evolutionary patterns. By integrating PacBio HiFi long-read sequencing with Hi-C chromatin conformation capture data, we obtained a highly contiguous genome assembly in which all sequences were successfully anchored to 33 pseudochromosomes. This chromosome number is consistent with previously reported cytogenetic karyotype analyses of *H. hodgsoni* [52], supporting the structural accuracy of the assembly at the chromosomal scale. Multiple independent quality assessments further confirmed the reliability of the genome assembly, including high mapping rates and coverage of HiFi reads, a high consensus QV estimated by Merqury, and a high proportion of complete conserved genes identified by BUSCO analysis. Collectively, these results indicate that the assembled genome meets current standards for high-quality mammalian reference genomes and provides a robust resource for downstream gene annotation, comparative genomics, and gene family evolution studies.

Based on the chromosome-level genome assembly, we performed a comprehensive annotation of the *H. hodgsoni* genome to characterize its gene content and overall genomic structure. A total of 23,424 protein-coding genes were predicted, with average gene length and exon organization comparable to those of other mammalian species, indicating a largely conserved gene architecture. The majority of predicted genes could be functionally annotated using multiple public databases, further supporting the reliability of gene prediction. Repeat sequence analysis revealed that approximately 29.90% of the *H. hodgsoni* genome consists of repetitive elements, a proportion lower than that observed in some comparative species (such as *A. albiventris*) [76] but comparable to that of other small mammals. This result suggests that large-scale repeat expansion has not occurred in the *H. hodgsoni* lineage, reflecting lineage-specific differences in transposable element dynamics and genome evolution among rodents and related mammals. Overall, the *H. hodgsoni* genome exhibits a conserved structural framework accompanied by moderate lineage-specific variation.

Comparative genomic analyses indicated that gene family evolution in the *H. hodgsoni* lineage is generally conserved, although a subset of gene families shows significant expansion or contraction. Among these, OR gene families exhibited particularly pronounced expansion. OR genes represent one of the largest and most rapidly evolving gene families in mammals and play a central role in odor detection and environmental sensory perception [77,78]. Functional enrichment analyses demonstrated that significantly expanded gene families in *H. hodgsoni* were strongly enriched in biological processes and molecular functions related to olfactory perception, including OR activity, odorant binding, and detection of chemical stimuli involved in sensory perception of smell. Compared with model organisms such as *H. sapiens* and *M. musculus*, the lineage-specific expansion pattern of OR gene families in *H. hodgsoni* suggests that the olfactory system may have been shaped by specific ecological demands, such as nocturnal activity patterns [79], complex habitats [80], and reliance on chemical cues for foraging and environmental navigation [81]. Similar massive expansions of OR gene repertoires have been widely documented in other macrosmatic species, particularly among rodents and subterranean or nocturnal mammals that rely heavily on olfaction for survival [82,83]. This parallel evolutionary trend underscores that OR gene expansion is a common genomic mechanism facilitating ecological adaptation to low-visibility environments across diverse mammalian lineages [84]. In addition to sensory-related functions, several expanded gene families were also associated with epidermal development and keratinization, implying that coordinated genetic changes in sensory perception and integumentary structures may underlie key adaptive traits in this species.

Further chromosome-scale analyses revealed that OR gene family expansion in *H. hodgsoni* is not only reflected by increased copy number but is also accompanied by pronounced local clustering. A large proportion of OR genes were concentrated within a limited number of chromosomal regions, most notably forming dense gene clusters on chromosome 9. Within these regions, OR genes are tightly arranged with short intergenic distances, displaying genomic architectures characteristic of tandem duplication. This highly clustered distribution pattern suggests that tandem duplication represents a major evolutionary mechanism driving the expansion of OR gene families in *H. hodgsoni*. Tandem duplication can rapidly increase gene copy number over relatively short evolutionary timescales and provide genetic substrates for functional diversification and adaptive evolution [85,86]. Similar clustered expansion patterns of OR genes have been reported in other mammals, further supporting the widespread role of tandem duplication in shaping OR gene repertoires [82,84,87].

Taken together, the pronounced expansion of OR gene families and their highly organized clustered chromosomal architecture indicate that the evolution of olfactory-related genes may have played an important role in the adaptation of *H. hodgsoni* to complex environmental odor cues [84]. Nevertheless, it should be noted that the present study is primarily based on genome-level comparative analyses and does not directly address the physiological or behavioral functions of individual OR genes or gene clusters. In addition, while our structural analyses provide strong evidence for tandem duplication, nucleotide-level positive selection analysis for such massively duplicated, multi-copy clusters remains methodologically challenging and prone to false positives. Future studies integrating multi-omics approaches—including rigorous selection signature analyses, transcriptomic and epigenomic profiling—with functional assays and behavioral analyses will be essential to decipher the regulatory mechanisms and functional diversification of OR genes across diverse tissues and developmental stages. Such efforts will provide deeper insights into the biological significance of OR gene expansion and the broader adaptive evolution of sensory systems in *H. hodgsoni*.

Furthermore, the high-quality PacBio HiFi long-read sequencing data generated herein not only resolves the nuclear genome but also provides an excellent resource for future assembly and refinement of the *H. hodgsoni* mitochondrial genome (mitogenome), which will further facilitate phylogeographic and evolutionary studies.

## 5. Conclusions

In this study, we generated the first high-quality, chromosome-level reference genome for *H. hodgsoni* using a combination of PacBio HiFi and Hi-C technologies. Comprehensive genomic analyses revealed the expansion of olfactory receptor (OR) gene families driven by tandem duplications, providing novel insights into the genetic basis of sensory adaptation in this species. This genomic resource will serve as a valuable reference for future studies on the evolution, conservation, and ecological adaptation of porcupines and related mammalian taxa.

## Figures and Tables

**Table 1:** Summary of genome assembly and annotation of *Hystrix hodgsoni*.

Sequencing	*Hystrix hodgsoni*
PacBio Sequel II sequencing	
Raw data (Gb)	~158
Sequencing depth (×)	27
Number of reads	4,573,090
Mean read length (bp)	18,588
Hi-C sequencing	
Clean data (Gb)	~158
Sequencing depth (×)	100
Genome assembly	
Estimated genome size (Gb)	2.25
Assembly size (Gb)	2.36
Number of gaps	284
Contig N50 (Mb)	39.73
Scaffold N50 (Mb)	74.88
GC content (%)	41.70
Number of protein-coding genes	23,424
BUSCO completeness (%)	95.80
Average gene length (bp)	32,672
Average exons per gene	9.50

## Data Availability

The raw PacBio HiFi and Hi-C sequencing data reported in this paper have been deposited in the Genome Sequence Archive (GSA) of the National Genomics Data Center (NGDC), China National Center for Bioinformation (CNCB), under the accession number CRA037879 (BioProject: PRJCA057038; BioSample: SAMC6805945). The chromosome-level genome assembly and annotations are available in the Genome Warehouse (GWH) under accession GWHHODJ00000000.1. All genome assembly and annotation files are provided in standard formats. The chromosome-level assembly is in FASTA format, suitable for indexing by SAMtools or BWA. Gene annotations (GFF3), proteins, and CDSs are available for phylogenomic and functional analyses.

## Supplementary Materials

The following supporting information can be downloaded at: https://www.mdpi.com/article/10.3390/genes17060596/s1, Table S1: Estimation of the *Hystrix hodgsoni* genome size using various software with varying *k*-mer sizes and parameters, based on HiFi reads; Table S2: Summary of sequencing libraries used for genome assembly of *Hystrix hodgsoni*; Table S3: Statistics of Hi-C sequencing and mapping; Table S4: Statistics of chromosome-level genome assembly in *Hystrix hodgsoni*; Table S5: Assessment of genome assembly quality in *Hystrix hodgsoni*; Table S6: Summary statistics of non-coding RNA genes in *Hystrix hodgsoni*; Table S7: Statistics of transposon elements in the *Hystrix hodgsoni* genome; Table S8: General statistics of gene function annotation; Table S9: Summary of orthogroup inference statistics across five mammalian species; Table S10: Summary of significantly expanded and contracted gene families inferred by CAFE v5 (p < 0.05).
