# Supplementary material for: A Chromosome-Level Genome Assembly and Annotation of the Chinese Porcupine (Hystrix hodgsoni) Reveals the Expansion of Olfactory-Related Gene Families
Source: Genes (Basel). 2026 May 22;17(6):596. doi: 10.3390/genes17060596 (PMC13300390; doi:10.3390/genes17060596)
Supplement: Supplementary file 1 [file genes-17-00596-s001.zip › genes-4291095-supplementary.pdf]

Table S1. Estimation of the *Hystrix hodgsoni* genome size using various software with varying *k*-mer sizes and parameters, based on HiFi reads

**Methods**	***k*-mer**									
	**21**	**23**	**25**	**27**	**29**	**31**	**33**	**35**	**37**	**39**
	**Genome size (Gb)**									
GS v1 (CovMax 1k)	2.19	2.20	2.22	2.23	2.24	2.24	2.25	2.26	2.26	2.27
GS v1 (CovMax 10k)	2.19	2.20	2.22	2.23	2.24	2.24	2.25	2.26	2.26	2.27
GS v1 (CovMax 900k)	2.19	2.20	2.22	2.23	2.24	2.24	2.25	2.26	2.26	2.27
FindGSE	2.30	2.31	2.32	2.32	2.33	2.36	2.21	2.21	2.22	2.26
Formula	2.57	2.57	2.57	2.65	2.65	2.65	2.65	2.65	2.65	2.65
	**Heterozygosity (%)**									
GS v1 (CovMax 1k)	0.80	0.78	0.74	0.71	0.69	0.67	0.66	0.64	0.62	0.61
GS v1 (CovMax 10k)	0.80	0.78	0.74	0.71	0.69	0.67	0.66	0.64	0.62	0.61
GS v1 (CovMax 900k)	0.80	0.78	0.74	0.71	0.69	0.67	0.66	0.64	0.62	0.61

Table S2. Summary of sequencing libraries used for genome assembly of *Hystrix hodgsoni*

Library name	Tissues	Sequencing strategies	Insert size	Sequencing platform	Data size	Library function
PacBio HiFi	muscle	CCS	~8 kb	PacBio Sequel II	~158.5 Gb	Genome assembly
Hi-C	muscle	PE-150	-	Illumina NovaSeq 6000	~190 Gb	Genome assembly

Table S3. Statistics of Hi-C sequencing and mapping

**Statistics of Mapping**	
Clean Paired-end Reads	1,282,284,755
Mapped Read Pairs	859,203,808
Mapped Ratio (%)	67.01
PCR Dup Read Pairs	109,519,629
No-Dup Read Pairs	749,684,179
**Statistics of No-Dup Read Pairs**	
No-Dup Cis Read Pairs	453,769,888
No-Dup Cis Rate (%)	60.53
No-Dup Tran Read Pairs	295,914,291
No-Dup Tran Rate (%)	39.47
No-Dup Valid Read Pairs (cis >= 1kb + trans)	405,321,779
No-Dup Valid Rate (%)	54.07
No-Dup cis Read Pairs < 1kb	344,362,400
No-Dup cis Read Pairs >= 1kb	109,407,488
No-Dup cis Read Pairs >= 10kb	99,138,555

Table S4. Statistics of chromosome-level genome assembly in *Hystrix hodgsoni*

**Chr**	**No. of contigs**	**Length (bp)**	**No. of genes**
ChrX	13	135,492,361	1,095
Chr1	2	112,397,031	1,340
Chr2	4	93,119,600	1,625
Chr3	5	89,532,894	954
Chr4	7	85,495,823	1,327
Chr5	3	83,769,170	1,131
Chr6	6	83,741,905	1,147
Chr7	3	82,248,868	992
Chr8	5	80,973,692	1,284
Chr9	10	80238642	1,615
Chr10	3	79,536,744	1,721
Chr11	6	78,674,800	1,442
Chr12	2	77,304,647	810
Chr13	17	74,878,484	1,798
Chr14	10	74,844,988	1,237
Chr15	2	74,441,505	835
Chr16	4	74025281	1,265
Chr17	9	72,667,655	842
Chr18	4	72,523,842	755
Chr19	6	71,873,603	2,127
Chr20	3	69,477,392	1,147
Chr21	2	68,484,657	1,005
Chr22	10	68,139,283	835
Chr23	9	61,154,261	621
Chr24	4	61001333	706
Chr25	1	55,383,013	642
Chr26	3	50,763,886	813
Chr27	10	49,091,479	1,220
Chr28	2	47,101,566	1,293
Chr29	5	41,552,662	1,035
Chr30	1	39,993,539	806
Chr31	2	36,411,200	643
Chr32	3	32,392,917	806
Total No. of contigs	556		
Total length of contigs (Gb)	2,668,521,589		
Total No. of anchored contigs	176		
Total length of chromosome level assembly (Mb)	2,358,699,217		
Anchor rate (%)	88.39		

Table S5. Assessment of genome assembly quality in *Hystrix hodgsoni*

Item		Metric	Genome
Assembly completeness	Reads alignment	Mapping rate (%)	99.99
		Mean depth (X)	31.10
		Coverage rate (%)	99.99
Base accuracy	Merqury evaluation	Base pair QV	69.15
		Error rate (%)	1.21e-07
		K-mer completeness	92.48
Functional completeness	Gene	Complete BUSCOs (%)	98.30
		Complete and single-copy BUSCO (%)	96.70
		Complete and duplicated BUSCOs (%)	1.60
		Fragmented BUSC (%)	0.70
		Missing BUSCOs (%)	1.00

Table S6. Summary statistics of non-coding RNA genes in *Hystrix hodgsoni*

**ncRNA category**	**Number of genes**	**Total length (bp)**
rRNA	203	38,984
tRNA	14,270	1,012,123
miRNA	437	34,530
snRNA	2,949	337,555
Total	17,470	1,423,192

Table S7. Statistics of transposon elements in the *Hystrix hodgsoni* genome

	**Genome**		
	**Length (Mb)**	**% of repeat**	**% of genome**
Total repeat fractions	841.42	100.00	35.67
Class I: Retrotransposon	624.85	74.26	26.49
LTR Retrotransposon	164.79	19.58	6.99
Gypsy/DIRS1	0.87	0.10	0.04
Retroviral	162.61	19.33	6.89
Non-LTR Retrotransposon	460.05	54.68	19.50
SINEs	102.64	12.19	4.35
LINEs	357.41	42.48	15.15
Class II: DNA transposon	81.26	9.66	3.45
hobo-Activator	30.56	3.63	1.30
Tc1-IS630-Pogo	44.79	5.32	1.90
Unclassified	102.21	12.15	4.3
Tandem Repeat	27.59	3.28	1.17

Table S8. General statistics of gene function annotation

Type		Number	Percent (%)
Annotated	eggNOG	22,421	95.72
	GO	19,229	82.09
	KEGG	16,516	70.51
	InterPro	21,377	91.26
	PANTHER	21,210	90.55
	Pfam	19,879	84.87
	Uniprot	21,767	92.93
	NR	10,985	46.90
	AnimalTFDB	2,011	8.59
Unannotated	-	899	3.84
Total	-	23,424	100

Table S9. Summary of orthogroup inference statistics across five mammalian species

Category	*Hystrix hodgsoni*	*Atelerix albiventris*	*Cavia porcellus*	*Homo sapiens*	*Mus musculus*	Total
Number of genes	23,424	35,442	21,478	19,728	21,751	121,823
Genes in orthogroups	21,352	28,066	20,759	19,237	21,034	110,448
Percentage in orthogroups (%)	91.2	79.2	96.7	97.5	96.7	90.7
Unassigned genes	2,072	7,376	719	491	717	11,375
Percentage unassigned (%)	8.8	20.8	3.3	2.5	3.3	9.3
Orthogroups containing species	16,934	16,131	16,719	16,653	16,787	—
Species-specific orthogroups	99	985	50	47	95	1,276
Genes in species-specific orthogroups	367	3,127	578	229	1,104	5,405
Percentage species-specific genes (%)	1.6	8.8	2.7	1.2	5.1	4.4

Table S10. Summary of significantly expanded and contracted gene families inferred by CAFE v5 ($P < 0.05$)

Lineage (branch)	Expanded	Contracted	Unchanged
Hystrix hodgsoni	177	138	397
Atelerix albiventris	184	120	408
Homo sapiens	80	422	210
Mus musculus	165	346	201
Cavia porcellus	160	296	256
